# The Role of Warfarin and Rivaroxaban in the Treatment of Cerebral Venous Thrombosis

**DOI:** 10.7759/cureus.4589

**Published:** 2019-05-02

**Authors:** Muniba Fayyaz, Fakhar Abbas, Tooba Kashif

**Affiliations:** 1 Internal Medicine, Fatima Memorial Hospital, Lahore, PAK; 2 Cardiovascular Medicine, Stanford University School of Medicine, Stanford, USA; 3 Cardiology, Sindh Medical College, Dow University of Health Sciences, Karachi, PAK

**Keywords:** warfarin, rivaroxaban, cerebral venous thrombosis, oral anticoagulant, factor xa, factor x, vitamin k, dabigatran, apixaban

## Abstract

Cerebral venous thrombosis (CVT) is a rare complication of hypercoagulable states such as pregnancy, lupus anticoagulant syndrome, systemic lupus erythematosus, Crohn's disease, ulcerative colitis, malignancies, and the use of oral contraceptive pills. It most commonly occurs in young people, especially women, but can occur in the elderly as well. The signs and symptoms vary from focal neurological deficiencies to alteration in mental status. In this review, we compare the efficacy and safety profile of traditional anticoagulants heparin and vitamin K antagonists (warfarin) to novel oral anticoagulants, which include rivaroxaban, apixaban, dabigatran. The advantages of the new anticoagulants are their effectiveness, short half-life, oral intake instead of parenteral, and the decreased need for constantly monitoring prothrombin time (PT), activated partial thromboplastin time (APTT), and the international normalized ratio (INR). In this review, we discuss studies that demonstrate that these novel oral anticoagulants are effective and safe in treating cerebral venous thrombosis without many adverse effects when compared with traditional treatment options. There are also some case reports that point towards the effectiveness of newer agents; however, we need more studies with bigger samples to reach a conclusion in favor of new oral anticoagulants. The studies that have already been conducted can become the basis for conducting newer studies that can revolutionize the modern treatment for conditions like CVT.

## Introduction and background

Cerebral venous thrombosis (CVT) is a relatively uncommon and underdiagnosed form of stroke that accounts for 0.5%-1% of all strokes mostly occurring in young adults, especially in women. If not treated promptly, CVT can be life-threatening and potentially fatal [1]. Multiple risk factors have been associated in the causation of CVT, but in many cases the etiology is unclear. Some modifiable and non-modifiable risk factors include:

a) Prothrombic conditions like factor V mutation, protein C, S and antithrombin III deficiency and antiphospholipid antibody.

b) Inflammatory conditions like inflammatory bowel disease; Crohn’s and ulcerative colitis and their treatment with steroids are both associated with an increased risk of CVT. 

c) Pregnancy, puerperium, use of oral contraceptive pills (OCPs), and malignancies and other conditions with hypercoagulable states.

d) Sinusitis, trauma, surgery and certain procedures of the brain.

e) Certain medications, which include OCPs, tamoxifen, erythropoietin, and heparin [2].

## Review

Signs and symptoms 

Cerebral venous thrombosis can present with a wide range of symptoms from an underlying pathophysiological process involved with the obstruction of the cerebral venous sinuses. A thrombus in the cerebral venous sinus can lead to a decreased absorption of the cerebrospinal fluid (CSF) and consequently lead to increased venous and capillary pressure. This can further lead to the development of vasogenic edema and decreased perfusion, which ultimately leads to ischemic injury and disruption of the blood-brain barrier (BBB) with increased intracranial pressure (ICP) [3].

Clinical presentation

A prospective study in a Tunisian population was carried out between January 2009 and December 2012 and the clinical, radiological, and prognostic outcomes were recorded. The study showed that CVT can present with an acute (24%), subacute (64%), or chronic (12%) mode of onset. CVT can result in intracranial hypertension, focal neurological deficits, seizures, and encephalopathy. A headache is the most common presenting feature in 83% of the cases due to raised ICP, followed by seizures, focal motor deficits, papilledema, and mental status changes. The lateral and superior sagittal sinuses are most commonly involved [4].

Diagnosis

The American Heart Association and American Stroke Association (AHA/ASA) recommends imaging of the cerebral venous system in the diagnosis of suspected cases of CVT. Computed tomography scans are more widely and easily performed; however, magnetic resonance imaging (MRI) of the brain with T1, T2 weighted images combined with magnetic resonance (MR) angiography provides us with the best diagnostic modality for CVT. An elevated D-dimer assay can be frequently found in patients with CVT, but normal D-dimer levels do not rule out CVT [5].

Current guidelines for the treatment of CVT

Current Recommendations for CVT Treatment According to the AHA/ASA Guidelines include:

1. Testing for prothrombotic conditions, which include protein C, protein S, antithrombin deficiency, antiphospholipid syndrome, prothrombin G20210A mutation, and factor V Leiden. This can be helpful in the management of patients with CVT. Testing for protein C, protein S, and antithrombin deficiency is indicated two to four weeks after completion of anticoagulation therapy. There is a very limited value of testing in the acute setting or in patients who are taking warfarin. (Class IIa; Level of Evidence B).

2. In provoked CVT (associated with a transient risk factor), vitamin K antagonists may be continued for three to six months, with a target international normalized ratio (INR) of 2.0 to 3.0 (Class IIb; Level of Evidence C).

3. In an unprovoked CVT, vitamin K antagonists may be continued for six to twelve months, with a target INR of 2.0 to 3.0 (Class IIb; Level of Evidence C).

4. For patients with recurrent CVT, venous thromboembolism (VTE) after CVT, or first CVT with severe thrombophilia (ie, homozygous prothrombin G20210A; homozygous factor V Leiden; deficiencies of protein C, protein S, or antithrombin; combined thrombophilia defects, or antiphospholipid syndrome), lifelong anticoagulation can be considered, with a target INR of 2.0 to 3.0 (Class IIb; Level of Evidence C).

5. Physician consultation with expertise in thrombosis can be considered to assist in the prothrombotic testing and care of patients with CVT (Class IIb; Level of Evidence C) [6].

General considerations

The traditional therapy for venous thromboembolism includes an overlap treatment between parenteral low molecular weight heparin (LMWH) and the vitamin K antagonist warfarin. The treatment usually consists of LMWH for at least five days and is stopped when the anticoagulant response with warfarin is therapeutic, as monitored by the international normalized ratio INR of 2 and 3. Thereafter, warfarin is continued for a minimum of three months. At this point, the decision to stop or continue treatment depends solely on the balance between the risk of recurrence if warfarin is stopped and the risk of bleeding if it is continued. This management is safe and efficient but has its disadvantages, which include iatrogenicity, laboratory monitoring of the international normalized ratio (INR), and parenteral use of heparins and fondaparinux. Nowadays, the use of new oral anticoagulants (NOACs) whose action is specific for either thrombin or activated factor Xa is being considered. These agents include dabigatran, rivaroxaban, apixaban, and edoxaban. The mechanism of action of dabigatran includes the direct inhibition of thrombin (factor 2a) in a selective and competitive manner. Meanwhile, the mechanism of action of rivaroxaban, apixaban, and edoxaban is the competitive and selective direct inhibition of factor Xa. The activation of factor Xa links the intrinsic and extrinsic pathways in the coagulation cascade and acts as a rate-limiting step in thrombin formation. Hence its inhibition can prevent thrombin generation directly. Dabigatran and rivaroxaban have been approved for the treatment of venous thromboembolism in the United States. Currently, rivaroxaban is approved for the prevention of venous thromboembolism in patients undergoing elective hip and knee replacement surgery, for the prevention of deep venous thrombosis and pulmonary embolism. Also, rivaroxaban can be used in nonvalvular atrial fibrillation to prevent systemic embolism and stroke. The role of rivaroxaban in cerebral venous thrombosis has been discussed in a few cases [7-11].

The advantages and disadvantages of the new oral anticoagulants when compared with warfarin

The advantages of NOACs over vitamin K antagonists (VKAs), such as warfarin, are their high efficacy in preventing stroke in atrial fibrillation (AF) and non-valvular atrial fibrillation (NVAF), lower incidence of major bleeding, convenience of use, minor drug and food interactions, rapid onset and offset of action, short half-life and almost immediate activity and the rapid removal after withdrawal (influenced by the renal function). Due to their predictable pharmacokinetics (PK) and linear pharmacodynamics (PD) with a dose/response relationship and the anticoagulant effect, these agents are administered at a fixed dose and do not require laboratory monitoring. In contrast, some disadvantages include their higher cost, the absence of specific antidotes, and limited experience with these drugs. Furthermore, NOACs should not be used in patients with severe renal and hepatic disease (absence of validated monitoring test), patients with mechanical heart valves, individuals younger than 18 years of age, and elderly patients [8,11].

Case reports demonstrating the effectiveness of rivaroxaban in cerebral venous thrombosis

In patients diagnosed with any neurological conditions, achieving and maintaining an optimal INR is at times difficult. The Rocket trial stated that rivaroxaban has a decreased rate of most concerning bleeds: fatal bleeding and intracranial hematoma when compared to warfarin, which makes it useful for the treatment of cerebral venous sinus thrombosis. This was demonstrated well in a case report done on a 37-year-old woman who presented with right-sided weakness and headaches for two months. On her MRI scan, a defect at the left sinus transversus was present. In this patient, rivaroxaban was given at a dose of 15 mg twice daily. On the fifth day follow-up, the right-sided weakness was relieved. Thereafter, rivaroxaban was continued for 21 days, followed by 20 mg once daily. On the third month follow-up, there were no complications in this patient. Hence, this case demonstrated that the use of rivaroxaban has a potential role when treating cerebral venous thrombosis [12].

Another case demonstrating the role of rivaroxaban in cerebral venous thrombosis included a 50-year-old female who presented with headaches, weakness of the right lower extremity, fever, and bone pain after receiving treatment for acute lymphoblastic leukemia with pegaspargase. The pegaspargase caused thrombosis in the bone marrow and brain. On bone marrow examination and imaging, this patient had developed bone marrow necrosis and cerebral venous thrombosis. This patient received rivaroxaban for the treatment of cerebral venous thrombosis and resulted in a good outcome with the neurological symptoms resolved, and the imaging studies showed the dissolution of thrombosis and bone marrow without necrosis. Hence, this case demonstrated well that rivaroxaban is safe and convenient to use as an anticoagulant in venous thrombosis leading to thrombosis at different sites such as cerebral venous thrombosis [13].

Yet another case report is that of a 17-year-old girl with inflammatory bowel disease (IBD) who presented with a rare complication of cerebral venous thrombosis. Her symptoms consisted of a headache and vomiting. MRI with venography showed venous thrombosis in the cortical veins, superior sagittal sinus, right transverse sinus, and right internal jugular vein. The patient was started on intravenous heparin infusion followed by oral rivaroxaban daily, which later resolved her symptoms and the imaging revealed normal results. She then continued rivaroxaban therapy for six months and remained well without neurologic sequelae. This case is another example of rivaroxaban used in cerebral venous thrombosis preventing fatal complications [14]. Rivaroxaban was used in place of warfarin in a patient with antiphospholipid syndrome who presented with recurrent cerebral venous thrombosis. In this case report, the patient was given warfarin initially, which resulted in a hemorrhagic ischemic stroke. Later, warfarin was replaced by rivaroxaban and prednisone. This case concluded that rivaroxaban prevented thrombosis and the hypercoagulability caused by prednisone [15].

Patel et al. discussed in their article the use of these new anticoagulant agents in the treatment of cerebral venous thrombosis. They reported that the use of the new anticoagulants such as rivaroxaban, dabigatran, and apixaban are associated with an increased risk of bleeding but might offer an alternative therapy for thrombosis when compared with the traditional therapy of heparin and warfarin [16]. Feher et al. conducted a literature review in which they concluded that there is not enough evidence supporting the use of the new oral anticoagulants in cerebral venous thrombosis even though they have proven clinical benefit in pulmonary embolism, deep venous thrombosis, and non-valvular atrial fibrillation. They also mentioned that in past case series rivaroxaban and dabigatran have shown good results [17].

Clinical trials demonstrating the effectiveness of novel anticoagulants in the treatment of CVT

A European study conducted to compare the efficacy of oral anticoagulants (OAC) for patients with cerebral and venous sinus thrombosis (CVST) course of recanalization was assessed and its association with clinical outcome was compared with a follow-up magnetic resonance imaging (MRI) and modified Rankin Scale 0-1 and was found to be excellent. Ninety-nine patients with CVST were included in a retrospective study between January 1998 and September 2004. In the study 97% of patients were treated with OAC; the median time for OAC was seven months. The median time for partial recanalization (PRec) was four months and time for complete recanalization (CRec) was six months. Recanalization rates were high on patients treated with OAC with 57% achieving CRec, 29% achieving PRec and only 13% of patients who did not recanalize. The outcome was excellent at clinical follow-up in 91.8% of patients and 2% of patients died. However, no association was found between recanalization rates and clinical outcome [18].

Another study that compared the efficacy of factor X inhibitors to vitamin K antagonist in patients with CVT data was recorded between January 2012 and December 2013. The modified Rankin Scale was used to assess clinical severity, and recanalization was assessed by follow-up magnetic resonance angiography (MRA). Sixteen patients were included in the study, seven were treated with rivaroxaban. The overall outcome was excellent in 93.8% of patients, and all patients showed at least partial recanalization. Statistical significance was not found between the two treatment groups except the use of heparin bridge in patients treated with VKA (P=0.03). Hence, the study showed that factor Xa inhibitors have a similar clinical profile as compared with VKA. One patient in VKA and two patients in factor Xa inhibitors had minor bleeding complications that led to a reduction of dose. In young patients with CVT, rivaroxaban might be considered an alternative to VKA because of its known effect and metabolism [19].

We also examined a systemic research of six patients who were admitted for CVT in the stroke unit of a tertiary center in Rome, Italy between January 2010 and July 2014. Follow-up visits were scheduled at three and 12 months, with MRI to assess vessel recanalization. Four patients were initially anticoagulated with LMWH for four to seven days. Rivaroxaban 20 mg was started after the median dose of seven days of heparin in two patients; two other patients were first transitioned to acenocoumarin for 15 days in the first patient and three months in other, then finally to rivaroxaban once daily. Two other patients started on rivaroxaban from the start. No other antiplatelet drug was started in any patient. The results of this study showed an excellent outcome with a modified Rankin Scale 0-1 observed in 100% of patients. There were no bleeding complications. This was a small retrospective study; however, rivaroxaban showed similar clinical benefits like other OACs with a better safety profile [20].

A report of three cases of confirmed CVT presenting with typical features were treated acutely with heparin and then transitioned to apixaban and discharged safely. These patients tolerated apixaban well with no bleeding complications and on follow-up scan demonstrated resolution of thrombus and recanalization. Current guidelines do not recommend the use of factor Xa inhibitors for the treatment of CVT; however, apixaban could be considered a safe alternative for the treatment of CVT [21].

The use of oral anticoagulants in mechanical/bioprosthetic heart valves and valvular heart disease

The American College of Cardiology/American Heart Association/Heart Rhythm Society (ACC/AHA/HRS) and 2010 European Society of Cardiology (ESC) guidelines stated that for atrial fibrillation and valvular heart disease the conventional management is recommended. In those who have an atrial fibrillation and mechanical heart valves, vitamin K antagonists are indicated to prevent systemic embolisms. Anticoagulants should be based on the position and type of the prosthesis, with an INR maintained at at least 2.5 for mitral valves and at least 2.0 for aortic valves [22-23]. These guidelines also recommended that dabigatran should not be used in patients with atrial fibrillation and a mechanical heart valve. In 2014, the guidelines from the ACC/AHA for managing valvular heart disease recommended that direct oral anticoagulants not be used in patients with mechanical valve prostheses [24]. For bioprosthetic heart valves, there is not much evidence for the use of direct oral anticoagulants. However, a study showed that catheter ablation of atrial fibrillation with uninterrupted use of direct oral anticoagulants in patients with biological heart valves is safe [25]. Also, in patients with atrial fibrillation and an underlying valvular heart disease, a role of the direct oral anticoagulants as an alternative treatment to vitamin K antagonists can be seen in clinical trials. This is especially true for patients with atrial fibrillation and native aortic valve disease, tricuspid valve disease, or mitral regurgitation and a CHA2DS2-VASc score of 2 or greater. Recent valvular heart disease guidelines have incorporated these direct oral anticoagulants' indications, and therefore, an increased use of these agents in patients with atrial fibrillation and valvular heart disease is anticipated [26].

## Conclusions

To date, limited data is available on the safety profile of direct oral anticoagulant and factor Xa inhibitors for the treatment of CVT. However, direct acting OAC and factor Xa inhibitors are used safely for the treatment of deep venous thrombosis, pulmonary embolism, portal vein, and limb thrombosis as well. Current guidelines do not recommend the use of novel OACs for the treatment of CVT but the studies done so far can pave the way for larger clinical trials, which can produce more statistically safe significant data that can revolutionize the treatment of CVT, especially for remotely located people in developing countries who do not have access to tests like prothrombin time/activated partial thromboplastin time (PT/APTT) and INR.
